# Erysipelas: Treatment of, by Muriated Tincture of Iron

**Published:** 1852-08

**Authors:** 


*Erysipelas: Treatment of, by Muriated Tincture of Iron.* By HAMILTON BELL, Esq., F. R. C. S. E.

The author's intention being solely practical, he does not enter minutely into the pathology of erysipelas; but in order to explain, in some measure, the principle by which he was actuated, in employing a powerful tonic in a disease accompanied with so much fever and excitement, he records his opinion, that in inflammation, the capillary vessels having apparently lost the power of separating or electing the component parts of the blood which are necessary for functional purposes, and become, to a certain extent, inert tubes, a stream of blood is admitted for the circulation of which they are not calculated.

The treatment which he has adopted for many years, without failure in a single case, is the exhibition of the muriated tincture of iron. The first object is to have the bowels freely acted upon. If the disease be mild, fifteen drops are given every two hours; if more severe, twenty-five to thirty drops, persevered in night and day, whatever be the degree of fever or delirium. His only local applications are flour or cotton-wadding. Cases are appended in illustration.

The brother of the author, Dr. C. Bell, confirms the favorable report above given. He says he has given it in the idiopathic form, and in that consequent on injury, with equally good results, and he has found it useful at all ages, from infancy to advanced age.

So beneficial is this treatment in erysipelas, that Dr. C. Bell expresses his conviction that if boldly given in puerperal fever, many lives would be saved. —*Edinburgh Monthly Journal.*

